# Ciprofloxacin-Imprinted Polymers: Synthesis, Characterization, and Applications

**DOI:** 10.3390/polym18030388

**Published:** 2026-01-31

**Authors:** Ut Dong Thach, Minh Huy Do, Cong-Hau Nguyen, Utkarsh Kumar, Tongsai Jamnongkan

**Affiliations:** 1 Research Group in Pharmaceutical and Biomedical Sciences, Faculty of Pharmacy, Ton Duc Thang University, Ho Chi Minh City 700000, Vietnam; 2 Faculty of Chemistry, University of Science, 227 Nguyen Van Cu Street, Cho Quan Ward, Ho Chi Minh City 700000, Vietnam; domhuy@hcmus.edu.vn; 3 Vietnam National University Ho Chi Minh City (VNU-HCM), Linh Trung Ward, Ho Chi Minh City 700000, Vietnam; 4 Institute of Applied Technology and Sustainable Development, Nguyen Tat Thanh University, Ho Chi Minh City 700000, Vietnam; nchau@ntt.edu.vn; 5 Center for Advanced Science and Technology (i-CAST), National Chung Hsing University, Taichung 402, Taiwan; utkarsh218@gmail.com; 6 Department of Fundamental Science and Physical Education, Faculty of Science at Sriracha, Kasetsart University, Chonburi 20230, Thailand

**Keywords:** ciprofloxacin, imprinted polymer, solid-phase extraction, sensor

## Abstract

Ciprofloxacin, being a widely used antibiotic agent, has sparked growing interest in the field of molecularly imprinted polymers (MIP) for its selective recognition and removal. This review provides a comprehensive analysis of the recent advances in the synthesis and applications of ciprofloxacin-imprinted polymers. The examination of synthesis compositions for the preparation of these polymers includes thorough discussions on functional monomers, crosslinkers, initiators, and solid supports. Various imprinting techniques, including bulk, precipitation, co-precipitation, and surface polymerization, have been assessed for the fabrication of the imprinted polymers. Furthermore, the advancing imprinting techniques, encompassing nano-scale imprinting, multi-functional monomers, multi-template imprinting, and electrochemical imprinting, are also highlighted. Additionally, an extensive exploration of the diverse applications of these polymers is also presented. These applications encompass selective separation and removal of ciprofloxacin from environmental samples, visual and electrochemical detection in complex matrices, their use as a stationary phase for HPLC, drug release, and photocatalysis. This review offers valuable insights into the current advancements and potential future directions in the development of ciprofloxacin-imprinted polymers, emphasizing their importance in diverse analytical and environmental applications.

## 1. Introduction

Ciprofloxacin (CIP) is among the most widely used drugs for treating human and animal diseases caused by bacterial infections [1]. Beyond its medical application, CIP is also employed as a growth promoter in animal husbandry and aquaculture [2]. However, frequent administration and improper disposal of antibiotics potentially harm environmental and ecological systems. CIP residues can cause a range of adverse effects in humans, such as allergic reactions and chronic toxic effects [1,3]. Furthermore, the effectiveness of CIP against diseases is decreasing due to the overuse and misuse of antibiotics in human and veterinary medicine and the increased occurrence of antibiotic-resistant genes [4].

**Figure 1:**
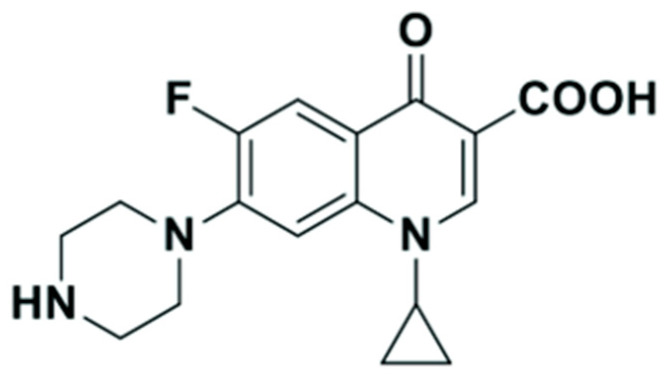
Chemical structure of ciprofloxacin.

CIP is a synthetic fluoroquinolone antibiotic structurally related to nalidixic acid (Figure 1). It is an antibiotic drug belonging to the fluoroquinolone class [5]. CIP is effective against both Gram-negative and Gram-positive bacteria, which makes it a versatile antibiotic for treating a wide range of infections such as bone-joint infections, skin infections in children, soft tissue infections, and respiratory infections [6]. However, excessive consumption of CIP has raised concerns about its potential presence in the environment. It also affects human health through the food chain and disturbs environmental and ecological systems [7]. Its accumulation in surface waters and soils can disturb aquatic ecosystems, damage soil microorganisms, and accelerate the development of antibiotic-resistant bacteria [8]. This pollution causes disturbances in aquatic ecosystems, damages soil microorganisms, and fosters the emergence of antibiotic-resistant bacteria, hence demonstrating substantial public health hazards [9]. Furthermore, CIP possesses toxicity towards both aquatic and terrestrial organisms, resulting in adverse effects on their behavior, reproduction, and development [10]. Additionally, CIP can accumulate in the organisms’ bodies, resulting in increased levels in predators, including humans. Efforts to mitigate antibiotic pollution include proper disposal of antibiotics, improved wastewater treatment, reducing unnecessary prescriptions, and raising awareness about antibiotic pollution [11]. Currently, researchers are interested in several issues related to CIP, including developing analytical methods, fabricating chemical sensors, developing removal techniques to treat CIP in water, and applying them in drug delivery [7,12,13,14].

Developing reliable analytical methods for the determination of CIP residue is urgently needed. Numerous analytical techniques have been developed to quantify CIP residues in various samples. Conventional approaches for detecting CIP involve the use of liquid chromatography in combination with UV-Vis or (tandem)mass spectrometry [14,15]. However, due to trace CIP levels in complex matrices, an initial sample pretreatment step such as liquid–liquid extraction, solid-phase extraction (SPE), or QuEChERS is often required for purification and enrichment. The efficiency of these pretreatments primarily depends on the properties of the absorbents used. However, commercially available adsorbents with low specificity may not be sufficient for the highly sensitive detection of CIP residue in complicated matrix components [16,17]. Additionally, these adsorbents may have several drawbacks that may limit their use for CIP monitoring, for example, high cost, excessive use of reagents, risk of contamination in different extraction steps, and interferences from other matrix components that possibly falsify the results. Therefore, an effective and simple sample preparation technique is needed to eliminate impurities and ensure the accuracy of analytical methods.

CIP sensors have gained considerable interest due to their high sensitivity, rapid response, ease of operation, and cost-effectiveness. Sensors typically consist of a receptor (recognition element) and a transducer (signal conversion element) [18]. The specificity of the receptor plays a decisive role in the identification of desired substances. A variety of sensing strategies have been demonstrated in the literature using naturally occurring molecular recognition features such as antibodies, DNA, antigens, and enzymes [19,20]. Although naturally occurring molecules have proven to be effective; however, some of these biomolecules are expensive and unstable. Consequently, the development of stable antibody-like materials with unique binding capabilities enables the successful selective detection of CIP residues.

Recently, molecularly imprinted polymers (MIPs), as selective sorbents used in SPE, have been known as an attractive alternative to overcome the previously mentioned limitations. Molecular imprinting is defined as a group of technologies used to create specific binding sites, usually using polymers, through a self-assembly process [21]. MIPs are created by the copolymerization of crosslinkers and functional monomers in the presence of template molecules via covalent, non-covalent, and hydrogen interactions [21]. After removing templates from MIP film, specific recognition sites can be obtained, which are complementary in terms of shape, size, structure, and functional groups of the templates [21]. The outstanding selectivity, recognition, and stable abilities of MIPs enable their use in various applications, such as selective adsorbents for sample pretreatment, sensing, CIP removal, drug delivery, etc. [22,23,24,25] Notably, conventional analytical methods, including solvent extraction and HPLC-UV, can be used for the quantifying high concentration of CIP in human plasma, with limit of quantification (LOQ) of 50 μg L^−1^ [15]. The MIP-SPE approach, in conjunction with HPLC-UV, is suitable for detecting trace amounts of CIP in complex samples, such as serum and plasma, with LOQ ranging from 0.074 to 0.113 μg L^−1^ [26]. Furthermore, MIP-based sensors have also attracted significant attention due to their unique template-specific recognition. Various CIP detection schemes integrating MIP layers electrochemical, surface plasmon resonance, and fluorescence have been reported in the past few years.

Beyond analytical pretreatment and sensing, molecularly imprinted polymers for ciprofloxacin have also been investigated in removal and delivery applications. For environmental remediation, CIP-MIPs are developed to selectively capture ciprofloxacin from complex aqueous matrices, with performance typically assessed in terms of adsorption efficiency, selectivity, and reusability [27,28]. In parallel, CIP-MIPs have been explored as drug delivery systems, where imprinting is used to modulate drug loading and release behavior, highlighting the versatility of CIP-MIPs across both analytical and functional application domains [29].

A comprehensive review by Madikizela et al. systematically summarized pharmaceutical pollutants in aquatic environments, with particular emphasis on analytical determination, occurrence, and environmental fate, including CIP among other fluoroquinolone antibiotics [30]. The present review differs in scope and focuses by concentrating specifically on CIP-centered materials, interactions, and applications, covering the period from their first reported synthesis by Caro et al. in 2006 [31] through early 2024. Meanwhile, we highlighted recent applications of CIP-MIPs for sample pretreatment, sensors, removal, and drug delivery. Finally, a summary, an outlook on this topic, and future directions for the topic are outlined.

## 2. Synthesis

### 2.1. Chemical Components Used for MIP Fabrication

#### 2.1.1. Functional Monomer

**Figure 2:**
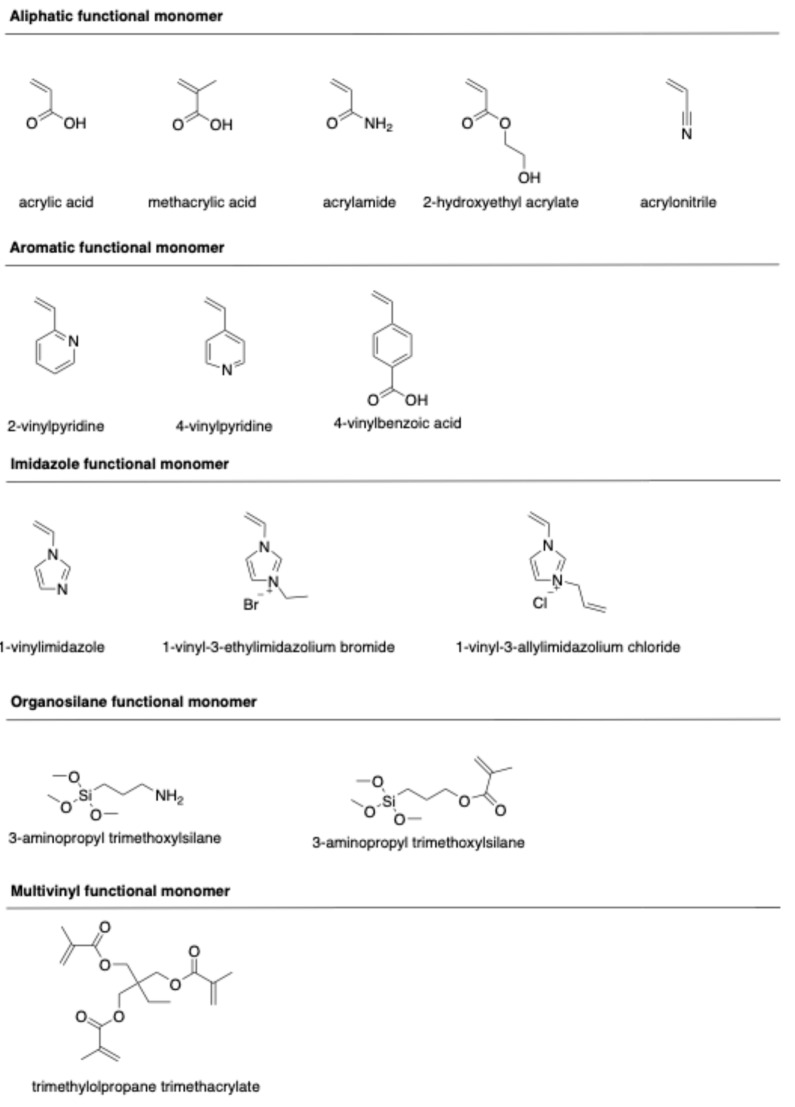
Chemical structure of functional monomers.

Molecular imprinting of ciprofloxacin is primarily governed by a combination of hydrogen bonding and electrostatic interactions between the template and functional monomers during pre-polymerization. Conventionally, functional monomers have been selected through trial-and-error tests based on experience and the chemical structure of CIP and functional monomers. The structures of typical functional monomers are illustrated in Figure 2.

Commonly used monomers for CIP imprinting include methacrylic acid (MAA), acrylic acid (AA), acrylamide (AM), 2-hydroxylethyl methacrylate (HEMA), 2-vinylpyridine (2-VP), and 4-vinylbenzoic acid (4-VBA). MAA is widely known as a universal functional monomer due to its unique characteristics. This monomer can interact with the template through hydrogen bond donor and acceptor interactions, making it highly suitable for ionic interactions. The dimerization property of MAA moderately enhances the imprinting effect [32]. Moreover, a high molar fraction of MAA results in a larger pore size in the polymeric network, thus enhancing the binding capacity of the resulting polymers [33].

Ionic liquid monomers have also attracted attention due to their chemical tunability, high thermal and chemical stability, nonvolatility, and excellent compatibility with both organic and inorganic species. Ionic liquid monomers, such as 1-vinyl-3-ethylimidazolium bromide (VEIMBr) [34] and 1-allyl-3-vinylimidazolium chloride (AVIMCl) [16], have been utilized to prepare CIP-imprinted polymers. The interactions between ionic liquid monomers and templates, including π-π interactions, electrostatic interactions, hydrogen bonding, dipole–dipole interactions, and hydrophobic interactions, contribute to enhancing the adsorption properties, selectivity, and water compatibility of the imprinted polymers.

Hydrogen bonds play a crucial role in the formation of complex pre-polymerization and specific rebinding sites during the imprinting and recognition process. However, these interactions can be disrupted by polar protic solvents, such as water or methanol, resulting in a reduction in the selectivity of the imprinted polymer. To overcome such challenges, multi-functional monomers have been employed. Various multi-functional monomer systems, including MAA/HEMA [35], [AVIM]Cl/HEMA [16], MAA/2-VP [36], and AA/1-VI [37], have been applied to synthesize CIP-imprinted polymers. The synergistic effect of these dual-functional monomers is responsible for the formation of imprinted polymers with high binding capacity, enhanced affinity, and superior selectivity [36].

**Figure 3:**
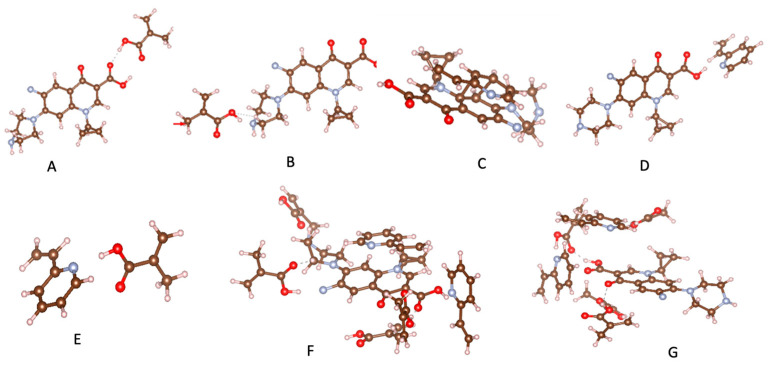
Most relevant interactions: (**A**) H---O for MAA-CIP, (**B**) H---N for MAA-CIP, (**C**) π-π stacking for 2-VP-CIP, (**D**) H---N for 2-VP-CIP, (**E**) H---N for MAA-2VP, (**F**) CIP-MIP, (**G**) CIP-NIP.

Computational simulation has emerged as an ideal approach to designing selective MIPs [38,39,40]. Sotomayor’s group employed semi-empirical simulation to assess twenty functional monomers and identify the most suitable for strong interaction with CIP [41]. Both computational calculations and practical experiments confirmed that MIPs synthesized with acrylamide monomer exhibited the highest specific selectivity factor and adsorption capacity. The same group also investigated the multi-intermolecular interactions of two functional monomers, namely AA and 1-VP, with CIP. MIPs synthesized using these two functional monomers demonstrated a faster binding of CIP to polymeric cavities and improved selectivity [37]. Similar observations were also reported in several publications [16,35]. Our group initially assessed functional monomer–CIP template interaction energies through static density function theory (DFT) calculations [42]. The findings demonstrate that hydrogen bonds play a significant role in MAA–CIP interactions. The configurations featuring hydrogen bonds between hydrogen atoms of MAA and oxygen or nitrogen atoms of CIP are notably the most stable, consistent with the observations made by Gómez-Pineda and Quiroa-Montalván [43]. For 2-VP, the most robust interaction observed is the π-π stacking between the aromatic ring of the template and the monomers, followed by hydrogen bonds. The hydrogen atom of the vinyl group of the monomer exhibits a relatively weak interaction with the oxygen and nitrogen atoms of CIP. Our research discovered that the hydrogen bond and π-π stacking interaction between the combination of MAA and 2-VP with CIP are the primary factors driving the development of selected rebinding sites in the synthesis of imprinted materials (Figure 3).

#### 2.1.2. Porogen

Porogens play a vital role as dispersion media and pore-forming agents during the polymerization process. The polarity of the porogenic solvent can significantly impact the bonding strength between functional monomers, as well as the morphology and adsorption properties of the polymer. Common solvents like toluene, dichloromethane, chloroform, acetonitrile, dimethyl sulfoxide, dimethyl formamide, methanol, ethanol, and dodecanol have been employed. Aprotic and low-polar organic solvents, such as toluene, chloroform, and acetone, are commonly used for CIP imprinting to achieve good imprinting efficiency. However, CIP-MIPs prepared in organic solvents may not perform well in aqueous media due to different issues such as “solvent memory”, different swelling ratios in water, and competitive sorption of water molecules. To enhance the water compatibility of MIPs, polar protic solvents like methanol, ethanol, and water can be used. Nevertheless, the hydrogen-bond donor and acceptor characteristics of polar protic solvents may disturb the interaction between the templates and functional monomers. The selection of the porogen is also limited by the solubility of CIP. To address these challenges, mixtures of solvents, such as toluene/methanol [27], toluene/methanol/dodecanol [34], chloroform/methanol [36], acetonitrile/methanol/triethylamine [44], dimethyl formamide/methanol [45], acetonitrile/water [37], ethanol/water/hydrochloric acid [46], and methanol/water [47], with appropriate volume ratios have been utilized to dissolve CIP and enhance the interaction between the templates and functional monomers, leading to improved selectivity and water compatibility of MIPs. The acetonitrile/water combination with a ratio of 7:3 (*v*/*v*) was found to be the most effective solvent for CIP imprinting. The CIP-MIP synthesized in an acetonitrile/water mixture exhibited an imprinting factor of up to 7.60 under optimal conditions [37]. In recent studies, room-temperature ionic liquids have been shown to accelerate the polymerization process, resulting in improved selectivity and adsorption capacity of imprinted organic polymers for trans-aconitic acid [48]. These ionic liquids exhibit great potential for the synthesis of novel CIP-imprinted polymers, characterized by improved selectivity and exceptional adsorption capabilities.

#### 2.1.3. Crosslinker

**Figure 4:**
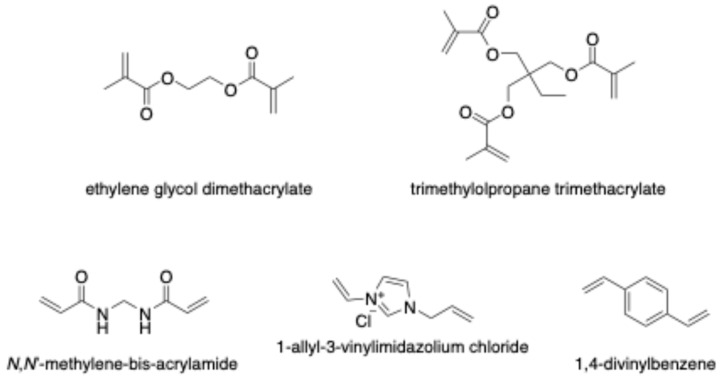
Chemical structure of crosslinker monomers.

The crosslinker plays a crucial role in fixing the functional monomer around the template and forming a highly cross-linked polymeric network after eliminating the template (Figure 4). The selectivity and binding capacity of MIPs are profoundly influenced by the type and amount of crosslinker used. Insufficient amounts of crosslinker can result in unstable mechanical properties due to a low degree of crosslinking, while excessive crosslinker may reduce the number of recognition binding sites per unit mass of MIPs [49]. Ethylene glycol dimethacrylate (EDGMA) has been commonly employed for synthesizing most reported CIP-MIPs. Additionally, Lui et al. utilized trimethylolpropane trimethacrylate (TRIM) to synthesize a CIP-imprinted polymer [50]. The use of 1,4-divinylbenzene (DVB) as a crosslinker facilitates the production of larger, uniformly sized particles with a narrower size distribution compared to other crosslinkers such as EDGMA or TRIM. A study by Rodríguez et al. proposed DVB as a co-crosslinker alongside EDGMA in synthesizing CIP-MIP. This combination yielded a material with a tightly controlled and well-defined particle diameter distribution, measuring 3.22 ± 0.03 μm, making it an ideal choice for online molecularly imprinted SPE applications [51].

In 2019, Zhu et al. presented a green and hydrophilic approach for developing a CIP-MIP synthesized in an aqueous solution [16]. In their method, they used a bifunctional monomer and crosslinker combination consisting of AVIMCl and HEMA. This combination enabled the formation of multiple hydrogen bonds between CIP and AVIMCl/HEMA, resulting in increased affinity. Additionally, the positively charged imidazolium ring of AVIMCl interacted with the negatively charged O atom in CIP through electrostatic interactions, while π-π interactions were potentially formed between the imidazolium cation of AVIMCl and the benzene ring of CIP. The incorporation of the ionic liquid as the functional monomer and crosslinker significantly enhanced the imprinted material’s affinity in aqueous media.

The synthesis of BiPO_4_@GO modified CIP-imprinted magnetic polymer (BiPO_4_@GO-MIP) utilized *N*,*N*-methylene bis-acrylamide as the crosslinker, showcasing remarkable selectivity towards CIP [52]. This imprinted polymer proved its effectiveness for both visual detection (using fluorescence study) and trace-level detection (via electrochemical study) of CIP in complex matrices. Moreover, the BiPO_4_@GO-MIP demonstrated successful applications in the separation and removal of CIP from complex matrices, with the added capability of completely degrading the separated CIP under UV-visible light.

#### 2.1.4. Solid Support

The addition of functional materials can be tailored for various purposes, including enhancing thermal and chemical stability, increasing in specific surface area and adsorption capacity of the proposed MIPs. Several common carriers have been successfully incorporated with CIP-MIPs, such as graphene oxide [34], carbon nanotube [53], ZnS [27], CdTe quantum dots [53,54], nanoparticle Fe_3_O_4_ [37], CoFe_2_O_4_@(poly-o-phenylenediamine)-Ag [55], and Au@chitosan nanoparticles [37], as well as mesoporous silica [56], TiO_2_ [57], magnetic fly-ash [46], yeast powder [35], stainless steel wire [26], polystyrene [45], polystyrene-co-divinylbenzene [16], and metal–organic framework (MOF) [58]. Selection of a solid support is based on the carrier’s properties, including high specific surface area, thermal and chemical stability, magnetic, electronic, and optical characteristics, and particle size distribution, to ensure suitability for the intended application.

### 2.2. Imprinting Techniques

#### 2.2.1. Bulk Imprinting

Bulk imprinting is considered the pioneering technique for synthesizing molecularly imprinted materials [31] (Table 1). The bulk form is well-suited for various applications in both academia [36,59] and commercial MISPE cartridges [60].

In 2006, Caro et al. reported the synthesis of the first CIP-imprinted polymer through bulk co-polymerization of MAA and EDGMA in dichloromethane [31]. The resulting MIP was then used to selectively extract CIP from urine samples using a two-step SPE procedure, combining a commercial Oasis cartridge and an MISPE cartridge in series. The urine extracts obtained after the two-step SPE procedure were relatively clean and could be directly injected into a mass spectrometer. In another study, Yan et al. proposed the synthesis of CIP-imprinted polymer in a water-containing system, specifically using methanol/water (7:3, *v*/*v*), with MAA as the functional monomer and EDGMA as the crosslinker. The obtained MIP was applied as a specialized chromatographic stationary phase for the selective separation of CIP from human urine samples [47]. Additionally, Kamel and Oliveira developed a biomimetic CIP sensor by incorporating bulk CIP-imprinted polymers into a polyvinyl chloride (PVC) matrix. The resulting sensors demonstrated high selectivity and sensitive response to the template in an aqueous system [60,61].

The bulk polymerization technique has several intrinsic limitations, including the loss of polymer particles during crushing and sieving steps, the destruction of specific cavities, low adsorption capacity, low kinetic adsorption, and unsatisfactory chromatographic resolution. To overcome these limitations, CIP monolithic imprinted materials have been developed, eliminating the need for post-polymerization steps. The CIP-imprinted monolithic columns are prepared by in situ polymerization within the confines of a stainless-steel column tube [62], pipette-tip [59], or SPE empty column [34].

#### 2.2.2. Precipitation Imprinting

By adjusting precursor ratios and polymerization conditions in bulk imprinting, such as the porogen volume and solvent polarity, MIPs with aggregated spherical microsphere particles (100–300 nm) can be obtained [23,41,59,63,64]. With this approach in mind, Turiel et al. prepared CIP-imprinted polymers using precipitation polymerization and packed them into a stainless-steel column (50 mm × 4.6 mm) for screening fluoroquinolones (FQs) in soil samples. They explored two different functional monomers (MAA, 4-VP) and four types of porogens (dichloromethane, methanol, acetonitrile, or toluene) and found that the MIP prepared in methanol using MAA as the monomer exhibited the best performance. Under the optimal condition, the developed MIP LC-UV method displayed satisfactory recoveries of over 93% and a limit of detection (LOD) of 0.21 μg g^−1^ [63]. In another study, Prieto et al. developed a microextraction by packed sorbent (MEPS) device based on precipitation CIP-imprinted polymers. The MEPS method extraction performance was evaluated using the LC-MS/MS method, indicating efficient analysis of target compounds in wastewater samples at ng L^−1^ levels, with a recovery of 115% and LOD of 0.8 ng L^−1^ [65].

#### 2.2.3. Co-Precipitation Imprinting

**Figure 5:**
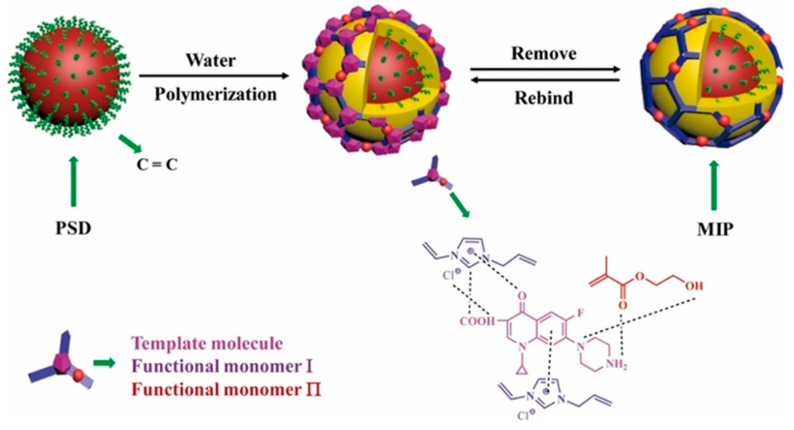
Schematic representation of the synthesis pathway for the ciprofloxacin-imprinted polymer by co-precipitation methods [16].

Co-precipitation polymerization of MIP components with other functional materials offers a versatile approach to creating composite imprinted polymers with promising advantages. These composite MIPs can be customized for various purposes, such as functionalization, improving physical and chemical stability, increasing specific surface area, and enhancing adsorption capacity [66]. Various common materials, including zinc sulfide (ZnS) [27], TiO_2_ [46,57], carboxylic functionalized multiwall carbon nanotubes (MWCNTs) and quantum dots (QDs) [53], stainless steel wire [26], poly(styrene-*co*-divinylbenzene) (PSD) [16], polystyrene microparticles [44], and chitosan gold nanoparticles [46], have been successfully incorporated with MIPs to modify the performance of composite imprinted polymers. These composites have exhibited exceptional adsorption and extraction performance, including rapid kinetic sorption, high capacity, and selective sorption [16,26]. For instance, TiO_2_ MIP composites showed selective degradation photocatalytic activity for water treatment applications [46,57]. Additionally, microcomposite or nanocomposite MIP particles with additional optical or electrochemical properties demonstrate the potential to develop biomimetic sensors to detect CIP antibiotics [45,53]. For example, Zhu et al. proposed the use of PSD to synthesize CIP-MIP materials via a co-precipitation polymerization technique (Figure 5). Vinyl-functionalized PSD particles were employed in a copolymerization reaction using 2-hydroxyethyl methacrylate and 1-allyl-3-vinylimidazole chloride as functional monomers. After polymerization, CIP was eluted, leaving specific sites capable of selectively adsorbing the target analyte molecules [16].

#### 2.2.4. Emulsion Polymerization

Emulsion polymerization is a radical polymerization process occurring in a mixture of water, monomer, surfactant, and an initiator. This method is commonly employed to create polymers with high molecular weight and distinct characteristics for controlling the size and shape of polymer particles. Flores-Ramírez et al. utilized emulsion polymerization to synthesize spherical CIP-imprinted polymer particles, using MAA and lactic acid functional monomers [29]. The MIP emulsion containing MAA has an irregular morphology compared to its counterpart without the drug, showing spherical particles of various sizes of about 5 µm. The MIP using lactic acid as the monomer produced spherical particles of various sizes, with a diameter of around 1 µm. In comparison, the corresponding NIP had particles with diameters of 10 µm. Polymers prepared using the emulsion approach exhibit a higher level of crosslinking and consequently provide better control over the release process, compared to the co-precipitation method. The MIP synthesized with MAA utilizing the co-precipitation method showed a slower release rate compared to the emulsion technique. The results indicate that both the imprinting techniques and synthetic components affect the drug release kinetics of imprinted polymers.

#### 2.2.5. Sol–Gel Polymerization

Sol–gel is a polymer synthesis technique that produces polymers with excellent water compatibility. This process involves the conversion of a colloidal solution (called “sol”) into an integrated network (called “gel”), including liquid and solid elements. The transition from sol to gel involves hydrolysis and polycondensation reactions, leading to the formation of a continuous solid network that entraps the liquid phase. Sol–gel polymerization is a simple, controllable, and cost-efficient method for creating imprinted materials [67,68]. Hui et al. suggested the synthesis of CIP-releasing silicone hydrogel contact lens materials by using HEMA, AA, and (trimethylsiloxy)silane (TRIS) functional monomers [69,70]. Novel contact lenses designed for prolonged delivery of CIP could offer significant benefits in improving therapies for sight-threatening microbial keratitis. Mohajeri et al. developed CIP-imprinted hydrogels using thermal sol–gel polymerization, employing MAA as the functional monomer and HEMA as both the backbone and solvent monomer [71]. The optimal imprinted hydrogel showed the strongest affinity to CIP with effective control over drug release. The hydrogels can block damaging UV radiation without affecting the absorption of visible light or the clarity of lenses.

#### 2.2.6. Surface Imprinting

**Figure 6:**
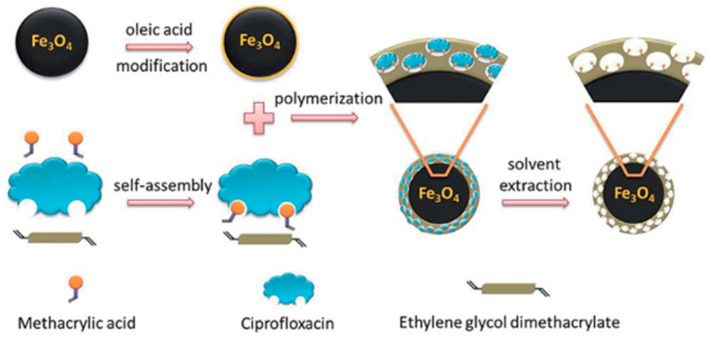
Schematic representation of the processes for the preparation of MIP-coated magnetic spherical nanoparticles by free-radical polymerization [75].

Micro-sized MIPs face several challenges, including difficulties in removing the template from interior binding sites, limited surface or near-surface binding sites, which result in low adsorption capacities, and restricted access to binding sites deep within the particles [66]. Moreover, the adsorption kinetics of template molecules onto bulk MIPs are often slow. The process generally occurs in two main steps: (i) mass transfer of the adsorbate from the sample solution to the polymer particle surface, and (ii) diffusion of the adsorbate into the specific rebinding sites [50,66]. To overcome these drawbacks and enhance mass transfer, rebinding capacity, and adsorption kinetics, surface imprinting techniques have been widely employed. In this approach, a thin layer of MIPs is covalently grafted onto the surface of different types of functional solid supports, such as silicone hydrogel [69,70], SiO_2_ nanoparticles [55,72,73,74], magnetic Fe_3_O_4_ nanoparticles [37,54,72,75,76], carbon nanotubes [53,77], yeast powder [35], and quantum dots [53,54]. The selection of support materials is based on their high specific surface area, thermal and chemical stability, and the presence of suitable functional groups for grafting polymerization. Several grafting techniques have been employed for anchoring the imprinted layer, including free-radical grafting [37,55,74,76], living polymerization via atom transfer radical emulsion polymerization [35], and silanization [53,54,72,73]. For example, Gou et al. prepared MIP@Fe_3_O_4_ nanoparticles by coating magnetic spherical nanoparticles via free-radical polymerization (Figure 6). Oleic acid binds to the surface of Fe_3_O_4_ nanoparticles through its –COOH group, while the double bond in the oleic acid chain reacts with the vinyl groups of the functional monomer, thereby forming an MIP layer on the surface of the Fe_3_O_4_ nanoparticles [75].

#### 2.2.7. Nano-Scale Imprinting

**Figure 7:**
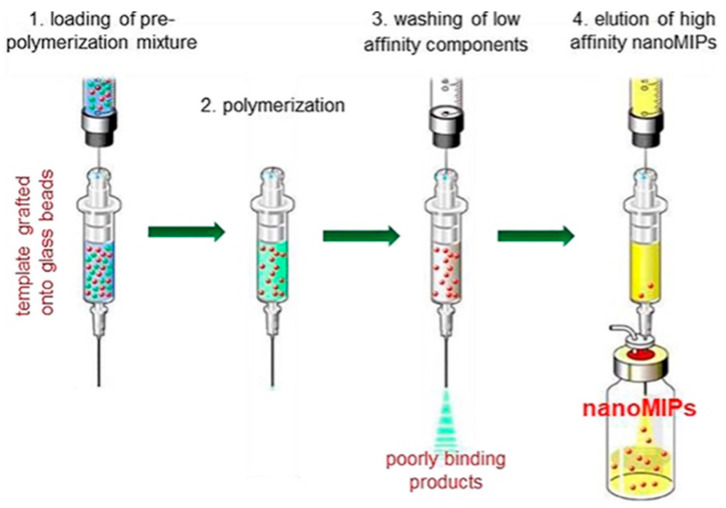
Schematic representation of the solid-phase synthesis method for the preparation of nanoMIPs [24].

Bulk and monolithic MIPs have limited applicability in sensing and separation techniques and present greater challenges for in vivo applications, including drug delivery and biomedical imaging. These are related to the characteristics of conventional MIPs, indicating irregular size distribution, low adsorption capacity, incomplete template elimination, and slow sorption kinetics. By combining nanotechnology and MIP, nanostructured MIPs (nanoMIPs) have been fabricated, which interestingly improved the properties of MIPs, compared to monolithic and bulk MIPs [66]. NanoMIPs could be synthesized using different polymerization techniques, such as precipitation imprinting [78], mini emulsion polymerization [20,79], surface or co-precipitation imprinting of nanoparticles [26,37,45,54,72,73,74,75,76], and solid-phase imprinting [24]. For example, the pre-polymerization mixture is first introduced onto glass beads functionalized with the template molecules, followed by polymerization on the solid support. Low-affinity and non-specifically bound polymers are then removed by washing, while the high-affinity nanoMIPs are selectively eluted and collected (Figure 7).

#### 2.2.8. Multi-Functional Monomers Imprinting

The competitive adsorption of water at specific binding sites through hydrogen bonding hinders the interaction between template molecules and the imprinting site, leading to a reduction in the selectivity of the imprinted polymers [16,47,80]. To overcome these challenges and enhance the adsorption capacity and selectivity of imprinted polymers, a strategy using multi-functional monomers has been developed. This approach allows multiple types of intermolecular forces with the template structure [81], resulting in improved molecular recognition and higher adsorption capacity and selectivity, especially for templates with strongly polar functional groups [81,82,83,84].

**Figure 8:**
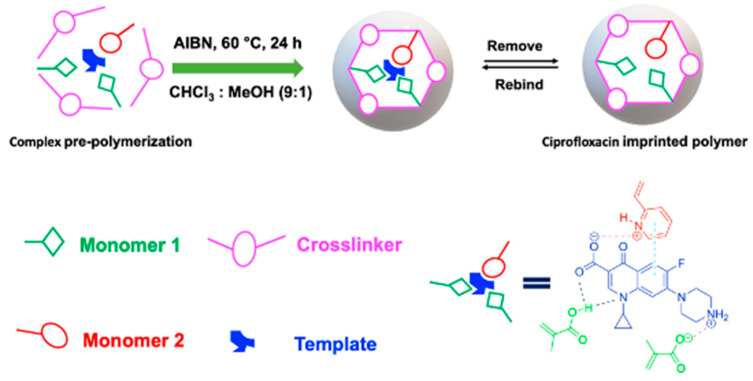
The use of dual-functional monomers for preparing CIP-imprinted polymer [36].

The pioneering work by Wang et al. introduced the synthesis of CIP-imprinted polymers using dual-functional monomers [35]. They employed a living co-polymerization approach with methacrylic acid and 2-hydroxyethyl methacrylate on the surface of functional yeast powder. The resulting yeast@MIPs exhibited a maximum static adsorption capacity of 18.48 mg g^−1^ for CIP, with an imprinting factor (IF) of 1.33, and achieved adsorption equilibrium within 60 min. This material demonstrated high affinity, selectivity, and efficient extraction performance of CIP from spiked shrimp samples for HPLC analysis. Similarly, Zhu et al. proposed a strategy using 1-allyl-3-vinylimidazolium chloride and 2-hydroxyethyl methacrylate as bifunctional monomers [16]. This approach enabled strong interactions between the obtained polymer and CIP molecules in an aqueous solution through hydrogen bonding, electrostatic, hydrophobic, and π-π-stacking interactions. The maximum rebinding capacity of CIP to MIP and NIP was 19.96 and 8.86 mg g^−1^, respectively. The remarkable selectivity and hydrophilicity of this imprinted polymer make it highly suitable as an adsorbent for SPE. Additionally, our group reported on the synergetic effect of dual-functional monomers (MAA and 2-VP) in CIP-imprinted polymer preparation (Figure 8) [36]. The MIP synthesized with these dual-functional monomers exhibited higher adsorption capacity and selectivity towards the template molecule, with favorable performance for SPE of CIP in aqueous media. Furthermore, Silva et al. synthesized magnetic CIP-imprinted polymers utilizing AA as a single functional monomer, and a combination of AA and 1-VP as two functional monomers [37]. The MIP synthesized by two functional monomers showed increased selectivity and a percentage of CIP adsorbed in the range of 87–99%. This material demonstrated superior performance for CIP in complicated matrices, e.g., milk and so on.

#### 2.2.9. Multi-Template Imprinting

To enhance the practical value of MIPs in simultaneous analysis, the multi-template imprinting technique has been employed. This approach involves two or more templates to prepare imprinted polymers, resulting in the creation of multi-specific rebinding sites within a single MIP format. Consequently, the simultaneous recognition and extraction of multiple target analytes can be achieved, offering a more versatile and efficient approach to addressing complex analytical challenges [66].

Lui et al. developed dual-template MIPs using levofloxacin and CIP as templates, MAA as functional monomers, and trimethylolpropane trimethacrylate as a crosslinker [85]. They established a method for simultaneously determining residues of ten fluoroquinolones in grass carp using high-performance liquid chromatography–ion trap mass spectrometry (HPLC-ITMS) in combination with molecularly imprinted polymer solid-phase extraction (MISPE). Recoveries ranged from 80.6% to 104.6%, with a relative standard deviation (RSD) of 8.6%. The limit of detection (LOD) was 0.11–0.25 µg kg^−1^, and the limit of quantification (LOQ) was 0.35–0.84 µg kg^−1^. This method is suitable for multi-residue analysis of fluoroquinolones in aquatic products. In a separate work, CIP and levofloxacin were employed as multi-templates to prepare an ionic-liquid-based, dual-MIP-coated graphene oxide SPE monolithic column. This column efficiently separated CIP and levofloxacin simultaneously from human urine, with recoveries of more than 93.8% under optimized conditions [34]. Additionally, Fan et al. prepared a multi-template MIP via surface molecular imprinting, using selected antibiotics (CIP, fleroxacin, enrofloxacin, norfloxacin, enoxacin, and lomefloxacin hydrochloride) as templates and mesoporous silica-modified magnetic graphene oxide as a support material. The developed SPE dispersive liquid–liquid microextraction (DLLME) HPLC method enabled the simultaneous analysis of trace target quinolones with spiked recoveries ranging from 89.67% to 100.5% and RSD of 3.59% to 7.12% [74].

#### 2.2.10. Electrochemical Imprinting

Electropolymerized MIPs involve polymerization reactions that happen on a solid electrode surface using electrochemical methods [86,87]. The preparation process of electropolymerized MIPs generally involves three steps: first, the self-assembly of functional monomers and target molecules occurs on the electrode surface through covalent or non-covalent interactions; second, polymerization is conducted on the electrode surface under galvanostatic, potentiostatic, or potentiodynamic conditions; and finally, target molecules are removed from the polymer film using physical, chemical, or electrochemical methods, leaving recognition sites on the surface of the substrate [88,89,90].

Electropolymerization has emerged as a highly preferred strategy for the synthesis of MIPs, especially in the context of chemical sensing, due to several advantageous aspects. Notably, this method offers a simple and rapid technique to fabricate a thin MIP film. Another advantage lies in the ability to control the film thickness by carefully selecting the electropolymerization parameters [86,90,91]. The effectiveness of the electropolymerized film is influenced by critical factors such as the type of monomer, electrode potential, and supporting electrolyte [87].

In the preparation of electropolymerized MIPs, electroactive monomers like pyrrole, *o*-phenylenediamine, aniline, *p*-aminothiophenol, 4-aminobenzoic acid, and phenylenediamine are commonly employed. However, only *o*-phenylenediamine, pyrrole, and 4-aminobenzoic acid have been reported on MIP synthesis for CIP by electropolymerization [58].

**Table 1:** Previously reported literature of ciprofloxacin-imprinted polymers.

Monomer	Porogen	Solid Support	Imprinting Technique	Application	Refs.
MAA ^1^	CH_2_Cl_2_	-	Bulk	SPE	[59]
MAA	MeOH:H_2_O		Bulk	HPLC	[23,47]
MAA	toluene	-	Bulk	SPE	[50]
MAA	MeOH:H_2_O		Bulk	Sensor	[61]
2-VP ^2^	MeOH:H_2_O		Bulk	Sensor	[61]
2-VP	ACN		Bulk	Sensor	[60]
MAA	ACN		Bulk	Sensor	[60]
AN ^3^	ACN		Bulk	Sensor	[60]
MAA	CHCl_3_	-	Bulk	SPE	
MAA	ACN		Bulk	In situ sampling	[17]
AA ^4^	ACN	-	Bulk	SPE	[41]
4-VBA ^5^	DMSO		Bulk	Adsorption	[17]
MAA/2-VP	CHCl_3_:MeOH	-	Bulk	SPE	[36]
MAA/AM	MeOH:H_2_O		Monolith	HPLC	[62]
[VEIM]Br ^6^	MeOH/toluene/dodecanol	GO	Monolith	SPE	[34]
MAA	MeOH	-	Monolith	pt-SPE^12^	[59]
MAA	HEMA		Hydrogel	Drug release	[71]
AA/TRIS ^7^	HEMA		Hydrogel	Drug release	[70]
HEMA ^8^/TRIS/AA	CHCl_3_		Hydrogel	Drug release	[69]
MAA	MeOH	-	Precipitation	SPE	[63]
MAA	MeOH	-	Precipitation	SPE	[64]
MAA	MeOH		Precipitation	SPE	[65]
MAA	ACN	Steel	Co-precipitation	SPE	[26]
MAA	MeOH/toluene	ZnS	Co-precipitation	Adsorption	[27]
AM, MAA or 4-VP ^9^	MeOH:H_2_O	TiO_2_	Co-precipitation	Photocatalyst	[57]
MAA	ACN: MeOH: Et_3_N	Fluorescent PS microparticles	Co-precipitation	Sensor	[92]
AVIMCl ^10^/HEMA	H_2_O	PSD	Co-precipitation	SPE	[16]
MAA	DMF:MeOH	Ch-AuNPs	Co-precipitation	Sensor	[45]
MAA/2-VP	ACN:H_2_O	PSD	Co-precipitation	SPE	[42]
Ti(OH)_4_	EtOH/H_2_O/HCl	Magnetic fly-ash	Xerogel	Photocatalyst	[46]
COOH@MWCNT ^11^/APTES ^12^	H_2_O	TGA-capped CdTeQDs	Sol–gel copolymerization	Sensor	[53]
APTES	H_2_O/EA	CdTe QD@Fe_2_O_4_	Surface	Sensor	[54]
MAA/HEMA	H_2_O/Tween-20	Yeast	Surface	DSPE^13^	[35]
MAA	H_2_O	Fe_3_O_4_	Surface	Sensor	[75]
APTES/TEOS ^13^	H_2_O	Fe_3_O_4_@m-SiO_2_	Surface	Sensor	[72]
MAA	DMSO	Vinyl-modified MMWCNTs	Surface	Sensor	[77]
TRIM ^14^	DMSO	Ag-POPD/CoFe_2_O_4_	Surface	Sensor	[55]
MAA	ACN	Fe_3_O_4_@MPS	Surface	Sensor	[76]
AM	EtOH	BrSiO_2_@pDA@PVDF	Surface	Sensor	[56]
AM	ACN	SiO_2_-FITC nanoparticles	Surface	Sensor	[73]
4-VBA	Toluene	SiO_2_	Surface	DLLME	[74,93,94,95]
AA/4-VI ^15^	H_2_O:ACN	Fe_3_O_4_@SiO_2_-C=C	Surface	Extraction	[37]

^1^ MAA: methacrylic acid; ^2^ 2-VP: 2-vinylpyridine; ^3^ AN: acrylonitrile, ^4^ AM: acrylamide, ^5^ 4-VBA: 4-vinyl benzoic acid, ^6^ [VEIM]Br: 1-vinyl-3-ethylimidazolium bromide, ^7^ TRIS: Tris(hydroxymethyl)aminomethane, ^8^ HEMA: 2-hydroxyethyl methacrylatem ^9^ 4-VP: 4-vinylpyridine, ^10^ AVIMCl: 1-allyl-3-vinylimidazole chloride, ^11^ COOH@MWCNT: COOH functionalized multi-walled carbon nanotubes, ^12^ APTES: 3-aminopropyltriethoxysilane, ^13^ TEOS: tetraethyl orthosilicate, ^14^ TRIM: 1-[2-(trifluoromethyl)phenyl]-1H-imidazole ^15^ 4-VI: 4-vinyl imidazole.

## 3. Characterization

### 3.1. Chemical Structure and Composition

The chemical structure and composition of imprinted polymers and their corresponding non-imprinted polymers can be determined using solid-state ^13^C CP-MAS (cross-polarization magic angle spinning) NMR spectroscopy, FT-IR, and elemental analysis [96]. Complete removal of the template is a crucial property for the practical application of MIPs in monitoring and quantitatively determining target analytes. The effectiveness of template removal in most reported MIPs is usually determined using FT-IR and UV-Vis or mass spectroscopy. More detailed analyses of FTIR patterns can be applied to evaluate the chemical structure of the synthesized CIP-MIP materials. To ensure thorough removal of CIP from the polymer, an ultrasonic bath is used for washing the polymer to confirm the effectiveness of template removal by determining the presence of CIP in the extraction solution using spectroscopy methods. Although this monitoring approach is indirect and may not detect trace residues of the template in the polymer network, it is a common method used in practice. Alternatively, an EDS comparative analysis and/or elemental analysis of MIP and NIP should be conducted before and after template extraction to confirm the complete elimination of the template from the imprinting process [97].

### 3.2. Surface Properties

The morphologies of MIPs are traditionally studied by using scanning electron microscopy (SEM), while transmission electron microscopy (TEM) and dynamic light scattering (DLS) are employed for characterizing nanoMIPs. To determine the specific surface areas of the polymers, nitrogen adsorption experiments are conducted, and the Brunauer–Emmett–Teller (BET) analysis is used. The pore size distributions are derived from the nitrogen isotherms’ desorption branch, employing the Barrett–Joyner–Halenda (BJH) method. Mesoporous and microporous parameters, such as external mesoporous surface, microporous volume, mesoporous volume, and total pore volume, are calculated from the linear segment of the α_S_-plot [98,99].

Thin-film MIPs can be characterized using various surface characterization techniques, including contact angles, atomic force microscopy (AFM), X-ray absorption fine structures, diffraction studies, and X-ray photoelectron spectroscopy (XPS) [97,100]. These techniques provide valuable information about the surface properties, topography, and composition of the thin-film MIPs.

### 3.3. Adsorption Properties

#### 3.3.1. Kinetic Adsorption

Kinetic adsorption plays an important role in the practical application of MIPs. Faster rebinding kinetics lead to improved mass transfer and extraction performance of these imprinted materials. Table 2 summarizes the adsorption kinetic parameters of reported CIP-imprinted materials prepared using various imprinting techniques. For instance, the adsorption kinetics of bulk MIP exhibited rapid binding in the first 2 h, covering approximately 82% of the total adsorption capacity. Subsequently, the rate of increase slowed down, and after 8 h, the adsorption process reached equilibrium with a total adsorption of 94% (Entry 1) [50]. This behavior can be attributed to the heterogeneous distribution of selective cavities at different depths within irregular bulk polymer particles. The adsorption kinetics on the outer surface particles were faster, while inward diffusion of the template through the pore canal was affected by mass-transfer resistance, leading to a decrease in binding kinetics.

**Table 2:** Adsorption kinetic parameters of CIP-imprinted materials synthesized from different imprinting techniques.

Equilibrium Time (min)	Q_e_ (exp)	Lagergren First-Order			Pseudo Second-Order			Imprinting Technique	Ref.
		q_e_ (mg g^−1^)	k_1_ (min^−1^)	R^2^	q_e_ (mg g^−1^)	k_2_ (mg g^−1^ min^−1^)	R^2^		
420	-	-	-	-	-	-	-	Bulk	[50]
8	-	8.66	0.605	0.9620	16.94	1.381	0.9998	Co-precipitation	[27]
180	19.26	2.74	-	0.1865	21.05	-	0.9912	Co-precipitation	[16]
60	11.67	11.37	0.3512	0.6232	11.62	0.07652	0.9558	Surface	[35]
60	29.38	18.63	0.054	0.9232	30.55	0.0067	0.9985	Surface	[73]
40	31.6	-	-	-	-	-	-	Surface	[77]
30	-	-	-	-	-	-	-	Surface	[37]
3	1.16	-	-	-	-	-	-	NanoMIP	[24]

In contrast, MIPs synthesized using advanced imprinting techniques such as co-precipitation, surface, and nano-scale imprinting generally exhibited faster adsorption kinetics. These techniques created a thin layer of imprinted polymer on the outer surface of a solid support or nano-sized MIP particles. The rapid template diffusion from the solution to the surface particles and their mass transfer within the thin layer of polymer facilitated the swift sorption kinetics of the template onto these imprinted materials.

To explore the binding mechanism in imprinted polymers, various kinetic models have been utilized [24]. These models include the Lagergren pseudo-first-order, the pseudo-second-order, and the Weber’s intraparticle diffusion kinetic model, represented by Equations (1), (2) and (3), respectively:

(1) $$q_{t}=q_{e}(1-e^{-k_{1}t}),$$

(2) $$q_{t}=\frac{q_{e}^{2}k_{2}t}{1+q_{e}^{2}k_{2}t},$$

(3) $$q_{t}=k_{id}t^{0.5}+C,$$

where *q*_*t*_ is the adsorbed amount at time *t*, *q*_*e*_ is the maximum adsorbed amount at equilibrium, and *k*_1_, *k*_2_, and *k*_*id*_ are the adsorption rate constants of the Lagergren pseudo-first-order model, the pseudo-second-order model, and the Weber–Morris intraparticle diffusion model, respectively.

As presented in Table 1, the pseudo-second-order model exhibited high regression correlation coefficient (R^2^) values ranging from 0.9558 to 0.9998 for all MIPs. This indicates that the pseudo-second-order model provided the best fit for the experimental data of adsorption kinetics. These results indicate that the chemical process was the rate-limiting step in the adsorption kinetics of the template on imprinted polymers [35].

#### 3.3.2. Effect of pH

**Figure 9:**
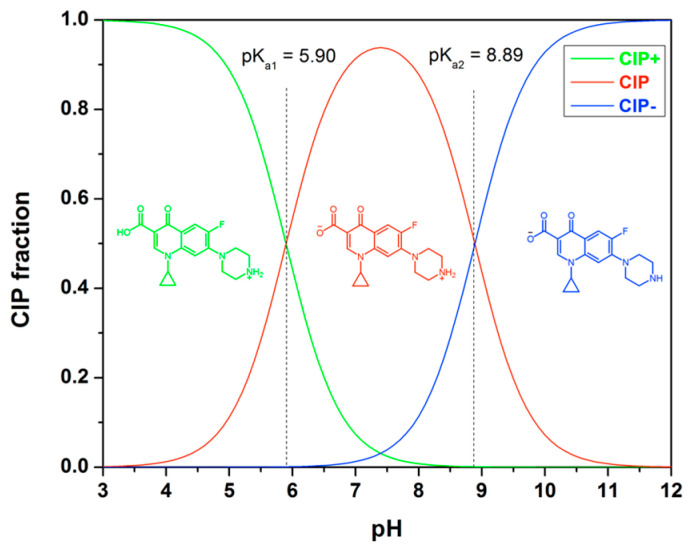
Molecular structure of ciprofloxacin and its ionic forms as a function of pH, calculated with pKa_1_ = 5.90 and pKa_2_ = 8.89.

Ciprofloxacin (CIP) exhibits different ionic forms in aqueous solution, as depicted in Figure 9. At below pH of 5.90, the cationic form CIP^+^ prevails. In the pH range of 5.90 to 8.89, CIP can exist as zwitterionic (CIP^±^). The dominant species within this range is the zwitterionic form CIP^±^, which results from a charge balance between the protonated secondary amine and the deprotonated carboxylic acid group on the piperazine group. Above a pH of 8.90, CIP predominantly exists as its anionic carboxylate form. The adsorption behavior of CIP is influenced by pH primarily due to the formation of different ionic species of the adsorbate and the surface charge of the adsorbent.

The selectivity and adsorption capacity of MIPs are influenced by the pH of the solution [36,42]. At pH values below 5, the adsorption process is limited. However, as the pH surpasses 5, the adsorption capacity of MIP4 significantly increases, reaching its peak at around pH 6.5. For pH levels exceeding 10, the sorption capacity decreases as the pH increases. This decline can be attributed to the competition between the adsorption of CIP^−^ and OH^−^ ions. Additionally, the weak sorption observed at extremely low and high pH ranges suggests a significant electrostatic repulsion between the ionic species of CIP and the surface of the adsorbent. It is noteworthy that the imprinted polymer specifically exhibits adsorption selectivity for CIP within the pH range of 5–7. This selectivity can be attributed to the imprinting process conducted in the weak acid medium of MAA. Therefore, for optimal selective extraction performance, it is important to conduct rebinding experiments within this pH range. The adsorption capacity and selectivity of imprinted polymers for CIP are influenced by the pH of the solution. Controlling the pH conditions is essential for achieving efficient and selective extraction of CIP using imprinted polymers.

#### 3.3.3. Adsorption Isotherm

The isotherm adsorption of ciprofloxacin on a ciprofloxacin-imprinted polymer refers to the relationship between the equilibrium concentration of ciprofloxacin in a solution and the amount of ciprofloxacin that is adsorbed onto the imprinted polymer at a given temperature. The adsorption isotherm provides valuable information about the binding affinity, capacity, and mechanism of the imprinted polymer for ciprofloxacin. Several commonly used isotherm models can be employed to describe the adsorption behavior, including the Langmuir and Freundlich models. These models make certain assumptions about the adsorption process and can help in understanding the adsorption mechanism.

The Langmuir isotherm assumes monolayer adsorption on a homogeneous surface and is described by the following equation:

(4) $$Q_{ads}\;=\;\;\frac{Q_{max}.C}{1\;+\;K.C}$$

where *Q_ads_* is the amount of ciprofloxacin adsorbed per unit mass of the imprinted polymer (mg g^−1^), *Q_max_* is the maximum adsorption capacity of the imprinted polymer (mg g^−1^), *C* is the equilibrium concentration of ciprofloxacin in the solution (mg L^−1^), *K* is the Langmuir constant related to the affinity of the adsorption (L mg^−1^).

The Freundlich isotherm, on the other hand, assumes multilayer adsorption on a heterogeneous surface and is represented by the following equation:

(5) $$Q_{ads}\;=\;K_{f}.C^{\frac{1}{n}}$$

where *Q_ads_* is the amount of ciprofloxacin adsorbed per unit mass of the imprinted polymer (mg g^−1^), *K_f_* is the Freundlich constant related to the adsorption capacity, *C* is the equilibrium concentration of ciprofloxacin in the solution (mg L^−1^), and *n* is the Freundlich exponent related to the adsorption intensity.

It is important to note that the choice of the appropriate isotherm model depends on the specific adsorption system and experimental conditions. Experimental data is obtained by measuring the equilibrium concentration of ciprofloxacin in solution before and after contact with the imprinted polymer and then calculating the amount of ciprofloxacin adsorbed. The data can then be fitted to the desired isotherm model to obtain the corresponding model parameters (Table 3). Overall, the isotherm adsorption study provides insights into the adsorption capacity, affinity, and mechanism of ciprofloxacin-imprinted polymers, which can be valuable for optimizing their use in various applications such as drug delivery, separation, or sensing.

**Table 3:** Adsorption isotherm parameters of reported CIP-imprinted polymers.

Functional Monomer	Solid	Q_exp._ (mg g^−1^)	Langmuir			Freundlich			Imprinting Technique	IF	Ref.
			Q_max_ (mg g^−1^)	K_L_ (L mg^−1^)	R^2^	K_F_ (L mg^−1^)	n	R^2^			
AM	-	93.00	0.09	0.0396	0.9950	24.50	0.5140	0.9670	Bulk	2.17	[41]
AA	-	74.00	0.07	0.0446	0.9930	30.30	0.3780	0.9030	Bulk	1.71	[41]
1-VI	-	76.00	0.07	0.0434	0.9960	29.10	0.3520	0.9190	Bulk	2.14	[41]
4-VBA	-	55.60	55.60	0.0400	0.9747	1.90	1.4000	0.9970	Bulk	1.16	[17]
MAA/2-VP	-	2.40	23.92	0.9487	0.7572	0.17	0.5873	0.8752	Bulk	1.66	[36]
MAA	ZnS	9.00	11.53	0.1270	0.9970	0.41	0.5500	0.9680	Co-precipitation	1.31	[44]
MAA	Steel	0.95	3.10 × 10^−3^	0.0031 × 10^−3^	0.9970	6.29 × 10^3^	1.85 × 10^3^	0.9440	Co-precipitation	6.06	[26]
AVIMCl/HEMA	PSD	19.96	19.96	0.1146	0.9764	2.62	1.9022	0.8503	Co-precipitation	2.25	[16]
MAA/HEMA	Yeast powder	19.61	19.61	0.0503	0.9919	3.17	2.8868	0.9749	Surface	1.36	[35]
APTES	Fe_3_O_4_@SiO_2_	33.18		-	-	-	-	-	Surface	1.69	[73]
VPD	SiO_2_	64.54	63.22	0.0059	0.9964	-	-	-	Surface	5.00	[56]
4-VBA	MGO@mSiO_2_	27.02	27.02	0.1380	0.9976	2.13	3.8190	0.9213	Surface	-	[75]
AA/1-VI	Fe_3_O_4_@SiO_2_	-	1.18	0.3770	0.9919	0.04	0.8600	0.9853	Surface	7.60	[37]

AM: acrylamide; AA: acrylic acid; 1-VI: 1-vinylimidazole; 4-VBA: 4-vinyl benzoic acid; MAA: methacrylic acid; 2-VP: 2-vinylpyridine; AVIMCl: 1-allyl-3-vinylimidazole chloride HEMA: 2-hydroxyethyl methacrylate; APTES: 3-aminopropyltriethoxysilane; VPD: vinyl pyrrolidone; IF: imprinting factor.

#### 3.3.4. Scatchard Analysis

In studies on molecule imprinting, the Scatchard Model was often used to evaluate the binding characteristics of MIP, and the Scatchard equation can be described as follows [101]:

(6) $$\frac{Q_{ads}}{C_{CIP}}\;=\;\frac{\left(Q_{max}\;-{\;Q}_{ads}\right)}{K_{d}}$$

where *K_d_* is the dissociation constant of the binding site, *Q_max_* is the maximum binding capacity of the binding site, *Q_ads_* is the binding capacity of ciprofloxacin, and *C_CIP_* is the equilibrium concentration of ciprofloxacin in the supernatant. In the Scatchard plot, the bound ligand concentration *Q_ads_* is plotted against the ratio of *Q_ads_/C_CIP_*.

For a non-imprinted polymer with homogeneous binding sites, the resulting graph is a straight line, and the slope and intercept of this line can provide valuable information about the binding properties. The slope of the Scatchard plot corresponds to the negative inverse of the dissociation constant (−1/*K_d_*). The association constant represents the strength of the receptor–ligand interaction, with higher values indicating stronger binding affinity. The intercept of the Scatchard plot on the *y*-axis represents the total number of binding sites (*Q_max_*). *Q_max_* represents the maximum amount of ligand that can be bound to the receptor. It provides information about the binding capacity of the receptor and can be used to estimate the receptor density or concentration. By analyzing the Scatchard plot, it is possible to determine the dissociation constant (*K_d_*), the number of binding sites (*Q_max_*), and potentially gain insights into the binding mechanism and the presence of different binding sites.

For imprinted polymers, the Scatchard plot does not exhibit a single linear relationship, indicating that the binding sites within the MIPs have varying affinities for CIP. Instead, the plot displays two distinct regions that can be considered as separate straight lines. This suggests that the binding sites can be categorized into two distinct groups, each possessing specific binding properties [47,102].

In cases where there are multiple binding sites or complex binding behavior, alternative methods such as nonlinear regression analysis may be required [50]. Therefore, a multi-point model was used to obtain the data fitted according to the multi-point model Formula (6) [87]:

(7) $$Q_{ads}\;=\;\frac{Q_{max1}.C_{CIP}}{K_{d1}+C_{CIP}}\;+\;\frac{Q_{max2}.C_{CIP}}{K_{d2}+C_{CIP}}$$

It is important to note that Scatchard analysis assumes a single class of identical, non-interacting binding sites. The validity of this assumption should be considered, as the actual binding behavior of the imprinted polymer may be more complex. Nonlinear regression analysis or other methods may be necessary if multiple binding sites or complex binding behavior are observed.

#### 3.3.5. Thermodynamic Adsorption

The thermodynamic adsorption refers to the study of the thermodynamic parameters associated with the adsorption process. These parameters provide insights into the energetics and spontaneity of the adsorption and can help understand the interactions between ciprofloxacin and the imprinted polymer. The thermodynamic parameters ΔG°, ΔS°, and ΔH° were employed using the following Equations [16,94]:

(8) $$∆G^{o}\;=\;-RTlnK_{L}$$

(9) $$lnK_{L}=\frac{{∆H}^{o}}{RT}+\frac{{∆S}^{o}}{R}$$

where Δ*G*°, Δ*H*°, and Δ*S*° are the change in free energy (kJ mol^−1^), enthalpy (kJ mol^−1^), and entropy (kJ mol^−1^ K^−1^), respectively; *R* is the ideal gas constant (8.314 J mol^−1^ K^−1^); *T* is the thermodynamic temperature (K); and *K_L_* is the Langmuir adsorption constant. The thermodynamic parameters of ciprofloxacin on several reported imprinted polymers are presented in Table 4.

Isothermal titration calorimetry (ITC) is a powerful technique used to determine the thermodynamic adsorption properties of a binding interaction, including the binding affinity (association constant), enthalpy change (∆*H*), and entropy change (∆*S*) [98,99,103]. Although ITC is commonly used to study biomolecular interactions, it can also be applied to the analysis of adsorption processes. ITC provides a direct measurement of heat changes associated with the adsorption process, allowing for accurate determination of the thermodynamic properties. Isothermal titration calorimetry (ITC) is highly recommended for studying the thermodynamic adsorption properties of molecularly imprinted polymers. By employing ITC, researchers can accurately characterize the energetics and mechanisms involved in the adsorption of target molecules by MIPs.

### 3.4. Determining of Imprinting Effect

The imprinting factor or selectivity of MIPs is commonly evaluated by comparing their binding capacity with that of their corresponding NIPs. However, it is essential to recognize that MIPs and NIPs possess different chemical and physical properties as they are synthesized under different conditions. Simply comparing the adsorption capacity of MIPs and NIPs may lead to misleading conclusions about MIP selectivity. A critical review contributed by Elizabeth N. Ndunda addresses this issue and explores the best approaches for determining the imprinting effect. The author investigates commonly applied methods for assessing the success of the imprinting process, considering the physical and chemical differences between MIPs and NIPs, and recommends approaches for determining key figures of merit in molecular imprinting. Dorko et al. showed that the IF alone is of limited value for assessing true MIP selectivity, as apparent selectivity enhancements may arise from nonspecific effects rather than imprinting [104]. Their results on MAA-based MIPs for propranolol demonstrated adsorption behaviors that differ from common literature assumptions. Given that ciprofloxacin also contains a protonatable amine, these findings caution against uncritical reliance on IF values when evaluating CIP-MIP selectivity. To accurately assess whether a memory effect has been created and whether the cavities in MIPs are specific to the template, thorough investigations using reliable methods are necessary [105].

Common methods for determining the selectivity of reported CIP-imprinted polymers include competitive adsorption experiments with other quinolone analogues (levofloxacin, norfloxacin, ofloxacin) and other drugs (pefloxacin mesylate, sulfamethoxazole, *N*-butylpyridinium chloride, sulfadiazine, ascorbic acid, and paracetamol) [16,37]. These experiments are carried out under static conditions using a batch adsorption process, with an excess adsorbate concentration to reach adsorption equilibrium. Under these conditions, the imprinted polymers demonstrated higher binding capacity and imprinting factor, while substantially lower adsorption amounts were obtained on NIPs. These results indicate that the presented MIPs have a higher affinity towards CIP and its quinolone analogues, revealing their imprinting effect and selective recognition ability.

In contrast, when other compounds with varying sizes, shapes, and functional groups were adsorbed onto MIPs and NIPs, the adsorption behaviors were found to be identical. However, it is important to note that the practical applications of MIPs involve dynamic conditions and low concentrations of trace target analytes. Therefore, it is highly recommended to perform selectivity studies of MIPs under dynamic column conditions and at trace analyte concentrations to better reflect their practical application and performance. This approach will provide more accurate insights into the specificity and effectiveness of the imprinted materials in practical scenarios.

## 4. Application

### 4.1. Solid-Phase Extraction

Conventional SPE adsorbents often lack the required selectivity as a limitation. The integration of MIPs as sorbent materials in SPE has become a highly promising application, offering specific recognition and enhanced selectivity for target analytes [87]. Molecularly imprinted solid-phase extraction (MISPE) has demonstrated successful applications in the extraction of CIP from diverse real samples, including environmental, food, biological, and pharmaceutical samples [26,59]. Table 5 provides an overview of various studies focusing on the extraction of CIP using MISPE. These MIP adsorbents for CIP can be prepared using different methods, such as bulk polymerization, precipitation polymerization, and surface imprinting [52,106]

Bulk polymerization is a commonly used method to prepare MISPE. For instance, Mirzajani et al. synthesized the MIP using CIP as the template, MAA as the monomer, and EGDMA as the crosslinker through thermal polymerization [106]. The resulting MIP was applied for selective SPE and quantification of CIP in biological fluids and pharmaceutical samples using HPLC-UV. The method exhibited a low limit of detection (LOD) of 0.028 μg L^−1^ and a recovery range of 98 to 101%. Additionally, the MIP prepared with MAA as the monomer and EGDMA as the crosslinker was utilized as a new sorbent for pipette tip SPE of CIP from human blood plasma, seawater, and tablet samples [61]. Under the optimal extraction conditions, the MIP pipette tip SPE showed a good linear correlation for CIP over a wide concentration range of 5–150 µg L^−1^, with a LOD of 1.5 µg L^−1^. Real applications for the determination of CIP in the samples resulted in satisfactory recoveries ranging from 86.9% to 99.6%.

In addition to bulk polymerization, other conventional methods, such as precipitation polymerization and surface imprinting, have been employed for the synthesis of CIP-MIPs [51,75,106]. For instance, Fan et al. prepared a unique type of MIP by using 4-vinylbenzoic acid grafted on mesoporous silica-modified magnetic graphene oxide as functional monomers, CIP as the template, EGDMA as the crosslinker, and AIBN as the initiator. This MIP was successfully applied to SPE and the detection of CIP in real water samples [74].

Furthermore, novel materials have been explored for the development of selective MISPE sorbents for CIP. Mirzajani et al. synthesized a MIP-coated core–shell nanosized magnetic sorbent (Fe_3_O_4_@SiO_2_@NH_2_-MIP) using the surface imprinting technique. This sorbent was used for simultaneous SPE and HPLC-UV determination of CIP in human fluid samples [106]. Ionic liquids have also been employed to prepare novel MIP SPE adsorbents for CIP. For instance, Ma et al. synthesized an ionic liquid-MIP-coated graphene oxide SPE monolithic column coupled with HPLC-UV. The monolithic column used 1-vinyl-3-ethylimidazolium bromide as a functional monomer for the selective MISPE of CIP from human urine [34]. The method showed good linearity with a high correlation coefficient of 0.9990. The recoveries of CIP in human urine samples ranged from 89.2% to 93.8%, and the LOD was 0.06 μg mL^−1^. In addition to laboratory-synthesized MIP sorbents, commercial MIP adsorbents have also been utilized for SPE to analyze CIP in various real samples. The SupelMIP^®^SPE–fluoroquinolones cartridge, a commercial product, has been applied for the MISPE to determine CIP and other fluoroquinolones in bovine kidney, honey, and milk [107].

The specific recognition ability of cavities on the surface of MIPs has also been harnessed as a stationary phase in chromatography, offering a new approach to the separation and purification of substances based on their specific interactions with the MIP matrix [88]. Although this concept shows great promise, there have been relatively few reports on the use of MIPs as stationary phases for the separation of CIP. For instance, Yun-Kai et al. investigated MIP layers formed on a conventional stainless-steel chromatographic column for the rapid separation and determination of CIP and enrofloxacin in eggs [108]. They studied the influence of different components, such as monomers, on the hydrophobicity, contractibility, selectivity, and binding capacity of the polymers. Under the optimal conditions, the MIP chromatographic stationary phase exhibited exceptional specificity, with a maximum binding capacity of 27.27 mg g^−1^. The mean recoveries of enrofloxacin and CIP from egg samples were found to be 103.3% to 115% and 89.3% to 95.1%, respectively. The LOQ and LOD were estimated as 8.3 ng g^−1^ and 27.7 ng g^−1^, respectively.

### 4.2. Stationary Phase for HPLC

At present, there is a growing preference for using MIP applications in analytical scale separation for SPE instead of HPLC. This is because MIP offers great extraction efficiency and requires a smaller volume compared to HPLC [109], resulting in the high effectiveness of CIP-imprinted polymer used in SPE. There are fewer reports on the use of MIPs as stationary phases for the separation of CIP [23,47,63]. The selective binding sites within the MIPs allow for efficient separation and analysis of CIP from complex mixtures. This application offers enhanced selectivity, sensitivity, and resolution in CIP analysis (Table 5). The recent studies of CIP-imprinted polymer in HPLC have been proven to be a validated method. The application of CIP-imprinted polymer in separation will be extensively exploited in the future.

### 4.3. Sensor

With their exceptional selective molecular recognition, MIPs have attracted significant attention with numerous applications, particularly in sensor technology. In recent years, various chemical sensors for detecting CIP have been developed by combining MIPs with advanced transducers, including electrochemical sensors, surface plasmon resonance, fluorescence, and cantilever-based sensors, as summarized in Table 6.

MIP-based electrochemical sensors for CIP detection have demonstrated significant potential due to their low cost, portability, high sensitivity, and ease of operation [111]. Among the various methods used for MIP polymerization, bulk imprinting and electropolymerization have become popular choices (Table 6). Additionally, the incorporation of supporting materials such as nanoparticles, metal–organic frameworks, and multi-walled carbon nanotubes (MWCNTs) with MIPs has been employed to enhance mechanical and electrical features. Several MIP-based electrochemical sensors have been developed for CIP detection.

One such sensor, developed by Bagheri et al., utilized differential pulse voltammetry to detect CIP in human serum and urine samples [78]. The CIP MIP was synthesized using a methacrylate functional monomer and EGDMA cross-linker. To amplify the signal of the sensor, a Fe_3_O_4_-MWCNTs nanocomposite was incorporated as a substrate layer during the MIP preparation using bulk imprinting. With the LOD of 0.0017 µM and a linear detection range of 0.005–0.85 µM, this CIP MIP-based sensor demonstrates favorable potential for routine analysis of CIP concentrations in pharmaceutical samples and biological fluids.

Another electrochemical sensor employed an electropolymerized imprinted polymer using a 4-aminobenzoic acid electrolytic functional monomer, which was coupled with co-metal–organic frameworks to enhance sensor sensitivity [58]. Under optimal conditions, the Co-MOF-MIP sensor exhibited a good linear correlation for CIP in the concentration range of 0.5–150 μM, with the LOD of 0.017 μM. In real applications for the determination of CIP in pill samples, the sensor achieved satisfactory recoveries ranging from 99.05% to 102%.

Some CIP-MIP sensors utilize the generation of a detectable optical response when MIPs bind to CIP molecules. There are two types of optical MIP sensors developed for CIP detection: MIP affinity sensors (such as fluorescence, refractive index, and optical absorbance) used to detect analytes with inherent optical properties, and optoelectronic MIP sensors. Among these optical sensors, fluorescence and surface plasmon resonance (SPR) detection are commonly used for CIP detection due to low detection limits and ease of fabrication.

One example is the work of Huang et al., developing a novel and sensitive fluorescent optical fiber sensor for detecting CIP in environmental water samples [19]. In this approach, the MIP film was prepared using MAA as the functional monomer and DVB as the crosslinker. Polyethylene glycol diacrylate hydrogel was incorporated into the MIP as a supporting material. After UV photo-polymerization and the removal of template molecules, the CIP-MIP fluorescence exhibited a good linear correlation in the concentration range of 10–500 μM, with high sensitivity and the LOD of 6.86 μM. Additionally, SPR sensors have shown great potential when combined with MIPs, as they can selectively monitor the real-time and label-free interaction of molecules, providing valuable information for CIP detection.

Luo et al. developed a CIP MIP using itaconic acid and EGDMA as functional monomer and crosslinker, respectively. The resulting MIP film was then coated on an SPR chip using the drop coating technique. This MIP SPR sensor demonstrated the capability to detect CIP within a linear range spanning from 10^−5^ to 10^−1^ µM, exhibiting excellent repeatability and long-term stability [110].

In this fluorescent sensing approach, the MIP is integrated into a hydrogel matrix positioned at the sensing region of the optical fiber, where it serves as a selective recognition layer. Upon exposure to the sample, ciprofloxacin is preferentially rebound by the imprinted sites, leading to its local preconcentration in close proximity to the fiber tip. The resulting enhancement of the fluorescence signal arises from this selective accumulation, while the polymer matrix itself provides mechanical support and optical transparency, thereby enabling sensitive and selective detection [112,113,114].

During template rebinding, electrochemical impedance spectroscopy (EIS) typically shows an increase in electron transfer resistance (R_ct_), which can be attributed to the occupation of imprinted cavities by ciprofloxacin and the resulting hindrance of redox probe diffusion and interfacial charge transfer. This change in R_ct_ provides indirect evidence of successful target recognition at the MIP-modified electrode surface. The magnitude of the resistance change depends on factors such as MIP layer thickness, porosity, and binding site accessibility, and thus offers insight into both the sensitivity and kinetic limitations of CIP-MIP-based electrochemical sensors.

Selectivity toward ciprofloxacin should be assessed not only against structurally related antibiotics but also against common biological interferents such as ascorbic acid and uric acid, which are abundant in clinical samples and may cause nonspecific responses. Evaluating interference from these species is therefore essential for sensor validation. Reusability and stability are also critical parameters, as stable sensor responses over multiple binding–regeneration cycles indicate robust imprinted sites and good long-term performance of the MIP film [115].

### 4.4. Applications of CIP-MIP as Adsorbents for CIP Selective Isolation

MIPs possess excellent properties, including chemical stability, high thermal resistance, remarkable affinity, and selectivity [116,117]. These advantageous characteristics have led to their widespread application as efficient adsorbents for the removal of CIP [117]. However, despite these promising features, there have been relatively few studies on the use of MIPs for CIP removal. Table 7 provides a summary of the research related to CIP removal using MIP materials.

Liu et al. successfully synthesized MIPs for the selective adsorption of CIP from aqueous solutions [51]. The material was prepared using the bulk imprinting technique, with CIP as the template, MAA as the functional monomer, EGDMA as the crosslinker, and AIBN as the initiator. In their study, they investigated the CIP/MAA/EGDMA ratio and contact time to optimize the adsorption efficiency of the prepared adsorbents. The resulting MIPs exhibited excellent affinity and selectivity toward CIP in aqueous samples, with an adsorption capacity of 29.4 mg g^−1^ at 25 °C.

Although conventional MIPs offer advantages in removing aqueous CIP, such as low cost, ease of preparation, and fast sample processing, they also come with certain limitations that hinder their broader applications. One significant drawback is the small particle size of these MIPs, which hinders adsorption in aqueous samples and limits their practical applications. To address these shortcomings, researchers have turned their attention to combining the molecular imprinting technique with nanostructured materials, which has gained significant interest lately. Various nanostructures, such as ZnS, ZnFe_2_O_4_ nanoparticles, and Bi(PO_4_)@GO, have been employed as support materials in preparing MIPs for CIP removal.

For instance, Tan et al. reported the preparation of MIP nanocomposites functionalized with ZnS nanoparticles for advanced CIP removal from aqueous samples [27]. These MIP nanocomposites exhibited exceptional selectivity and high removal efficiency, with adsorption capacity ranging from 7.67 to 8.65 mg g^−1^. In another study, Kumar et al. developed composite fluorescent BiPO_4_@GO-based magnetic MIPs for specific recognition and fluorescent quantification of CIP in real samples [52]. The resulting MIPs demonstrated high recognition selectivity and good adsorption capacity for CIP in milk, whole blood, and blood serum samples. Recent studies have demonstrated the synthesis of nanocomposite CIP-imprinted materials as effective and selective adsorbents for removing CIP in aqueous media [28,118,119]. By incorporating nanostructured materials into the MIP synthesis, these studies have shown promising results in overcoming the limitations of conventional MIPs and enhancing their performance in CIP removal.

### 4.5. Drug Delivery

Molecular imprinting approaches have effectively prolonged drug release duration in vivo for therapeutic molecules [120]. Hui et al. created an innovative silicone hydrogel by utilizing the imprinting approach to prolong the release duration of the fluoroquinolone antibiotic CIP [70,71]. The imprinted materials demonstrated sustained release for nearly 8 h, considerably longer than non-imprinted controls. Novel contact lenses designed for prolonged CIP delivery could therefore offer significant advantages in improving therapies for sight-threatening microbial keratitis. In a similar study, Kioomars et al. synthesized a CIP-imprinted hydrogel for use as a soft contact lens material [72]. The imprinted hydrogels showed effective antibacterial activity against *P. aeruginosa* and *S. aureus* strains isolated from patients’ eyes. Recently, Flores-Ramírez et al. synthesized CIP-imprinted polymers utilizing emulsion, bulk, and co-precipitation polymerization methods [29]. The material obtained by co-precipitation with MAA exhibited the highest adsorption capability for CIP. MIP-mediated CIP release demonstrated potent antibacterial action against *S. aureus* and *E. coli*, with MIC values ranging from 0.016 to 0.125 mg L^−1^ and 0.004 to 0.028 mg L^−1^, respectively. The polymers were tested on Human Dermal Fibroblasts (HDFs) cell lines, which validated their safety as materials and showed an increase in cell survival above 100%. The CIP-MIPs possess advantageous properties for use in pharmaceutical delivery systems and for use in wound dressings (Table 8).

## 5. Conclusions and Perspectives

In conclusion, numerous imprinting techniques have been successfully employed to prepare ciprofloxacin (CIP)-imprinted polymers, each contributing to enhanced material performance. The use of multi-functional monomer systems combined with optimized porogen compositions has been shown to significantly improve the selectivity and binding affinity of CIP-imprinted materials. Advanced imprinting strategies, including co-precipitation, surface imprinting, emulsion polymerization, and nano-scale imprinting, have demonstrated considerable potential in producing high-performance MIPs with well-defined recognition sites. The development of CIP-imprinted polymers has shown great potential in various applications, including drug delivery, selective extraction, and sensing. These MIPs exhibit high selectivity and affinity for CIP, enabling efficient detection and separation of this important antibiotic from complex matrices. The molecular imprinting process allows for the creation of specific binding sites that mimic the molecular structure of CIP, resulting in enhanced recognition capabilities. Moreover, the stability and versatility of MIPs make them attractive candidates for practical use in different fields. CIP-MIP has demonstrated promising results in various applications, and further research and development efforts can unlock its full potential. In solid-phase extraction and sensing applications, CIP-MIPs generally exhibit good selectivity and sensitivity, although matrix effects from complex samples and limited reusability can affect practical performance. For drug delivery, CIP-MIPs offer controllable loading and release behavior, but challenges remain in fine-tuning release kinetics and ensuring biocompatibility. In photocatalytic and removal applications, imprinting can enhance target recognition and degradation efficiency, while catalyst stability, regeneration, and effectiveness in real matrices remain key issues.

Various perspectives could be explored to advance the field of CIP-imprinted polymers. Further studies on polymer design factors, such as monomer selection, crosslinker proportion, and imprinting techniques, can improve the effectiveness of CIP-imprinted polymers. Optimizing the structure of the polymer and composition can enhance selectivity, binding affinity, and stability. It is crucial to conduct more research on CIP-imprinted polymers for diverse applications. Incorporating MIPs into various platforms such as biosensors, SPE cartridges, or drug delivery systems can maximize their effectiveness in targeted drug delivery, environmental monitoring, and clinical diagnostics. Expanding the identification capabilities of CIP-imprinted polymers to encompass other structurally similar antibiotics or related chemicals may be beneficial. Creating versatile MIPs capable of selectively identifying numerous analytes would expand their utility and overcome the difficulties presented by intricate sample compositions. Efforts should be focused on closing the disparity between laboratory-scale research and industrial-scale output. Scalable synthesis methods, cost-effective production, and full validation of performance are crucial for the commercialization of CIP-imprinted polymers.

## Figures and Tables

**Table 4:** Thermodynamic adsorption parameters of several MIP and NIP.

Monomer	Polymer	*T* (K)	*K_L_* (L mol^−1^)	∆*G*° (kJ mol^−1^)	∆*H*° (kJ mol^−1^)	∆*S*° (kJ mol^−1^ K^−1^)	Ref.
AVIMCl/HEMA	MIP	298.15	60,944.27	−27.31	−3.45	0.08	[16,77]
	NIP	298.15	71,531.80	−27.71	−3.66	0.08	
4-VBA	MIP	308.15	45.72	−10.25	−59.81	−0.1662	[77]
	NIP	-	-	-	-	-	

AVIMCl: 1-allyl-3-vinylimidazole chloride; HEMA: 2-hydroxyethyl methacrylate; 4-VBA: 4-vinylbenzoic acid.

**Table 5:** Application of MIP for the solid-phase extraction of CIP.

Monomer	Crosslinker	Initiator	Porogen	Imprinting Technique	Analytical Instrument	Recovery (%)	Sample	Detection Limit (µM)	Ref.
AA/4-VI	EDGMA	AIBN	ACN/H_2_O	Surface	HPLC-UV	87.0–95.0	Milk	-	[37]
HEMA	AVIMCl	AIBN	H_2_O	Co-precipitation	HPLC-UV	87.3–102.5	Lake water	0.11	[16]
MAA/HEMA			H_2_O/Tween-20	Surface	HPLC-UV	86.4	Shrimp	-	[35]
MAA	EGDMA	AIBN	CH_2_Cl_2_	Bulk	HPLC-MS	80–87	Urine	-	[59]
MAA	EGDMA	AIBN	MeOH	Bulk	UV-Vis	86.9–99.6	Seawater Tablet Blood plasma	1.50	[59]
MAA	EGDMA	AIBN	ACN	Bulk	HPLC-UV	98–101	Serum, plasma	0.028	[26]
MAA	EGDMA	AIBN	MeOH/H_2_O	Bulk	HPLC-UV	75.2–112.4	Seawater	0.0006	[23]
MAA/2-VP	EGDMA	AIBN	CHCl_3_/MeOH	Bulk	HPLC-UV	105	-	-	[36]
MAA/2-VP	EGDMA	AIBN	CHCl_3_	Bulk	HPLC-DAD	≥96	Milk	0.01–0.02	[95]
MAA/HEMA	DVB/EGDMA	ABDV	ACN	Precipitation	FLD-FLD	94–100	Real water	0.008–0.012	[51]
VTTSMGO@mSiO_2_	EGDMA	AIBN	Toluene	Surface	HPLC-PDA	95.2–99.7	Water	0.012	[74]
Fe_3_O_4_@SiO_2_@NH_2_	EGDMA	AIBN	ACN	Surface	HPLC–UV	94	Human fluid	0.0008	[106]
VEIMBr	EGDMA	AIBN	MeOH/Toluene/Dodecanol	Bulk	HPLC-UV	89.2–93.8	Urine	0.18	[34]

AA: acrylic acid, HEMA: 2-hydroxyethyl methacrylate; MAA: methacrylic acid, 2-VP: 2-vinylpyridine, VTTSMGO@mSiO_2_: magnetic graphene oxide@mesoporous silica modified with vinyl groups, VEIMBr: 1-vinyl-3-ethylimidazolium bromide, EDGMA: ethylene glycol dimethacrylate, AVIMCl: 1-allyl-3-vinylimidazolium chloride, DVB: divinylbenzene, AIBN: azobisisobutyronitrile, ABDV: 2,2′-azobis(2,4-dimethylvaleronitrile).

**Table 6:** Preparation of different chemical sensors for CIP detection.

Monomer	Porogen	Initiator	Crosslinker	Imprinting Technique	Detection Technique	Matrix	Linear Range (µM)	Detection Limit (µM)	Ref.
MAA/HEMA	H_2_O	NaHSO_3_/NH_4_S_2_O_8_	EGDMA	Bulk	Cantilever	Water	1.5–150.9	0.8	[20]
ITCA	EtOH/H_2_O	UV-LED	EGDMA	Bulk	Surface plasmon resonance	Water	0.0001−0.1	0.08	[110]
MAA/DVB	ACN	AIBN	EGDMA	Bulk	Fluorescent detector	Water	10–500	6.86	[19]
MAA/2-VP	MeOH/H_2_O	BPO	EGDMA	Bulk	Potentiometry	Fish/drug	10–27	3.31	[61]
MAA	DMF/MeOH	AIBN	EGDMA	Bulk	Cyclic voltammetry	Real water/milk/ drug	1–100	0.210	[44]
MAA	ACN/MeOH/TEA	AIBN	EGDMA	Bulk	Fluorescent detector	Fish	0.5–100	0.092	[44]
4-ABA	DMF	AIBN	EGDMA	Electropolymerization	Differential pulse Voltametric	Drug	0.5–150	0.017	[58]
AM	ACN	AIBN	EGDMA	Bulk	Fluorescent detector	Aquaculture water	0–250	0.004	[74]
MAA	DMSO/H_2_O	AIBN	EGDMA	Bulk	Differential pulse Voltametric	Drug/biological fluids	5–850	0.0017	[78]
ANI/ *o*-PDA	HCl/H_2_O	-	-	Electropolymerization	Differential pulse Voltametric	Real water	0.001−0.5	0.00005	[105]

MAA: methacrylic acid, HEMA: 2-hydroxyethyl methacrylate, ITCA: itaconic acid, DVB: 1,4-divinylbenzene, 4-ABA: 4-aminobenzoic acid, AM: acrylamide, CAN: acetonitrile, TEA: triethylamine, DMF: dimethylformamide, DMSO: dimethylsulfide, AIBN: azobisisobutyronitrile, BPO: benzoyl peroxide, EDGMA: ethylene glycol dimethacrylate, ANI: aniline, o-PDA: o-phenylenediamine.

**Table 7:** The summary of material based on MIPs for removing CIP.

Monomer	Crosslinker	Porogen	Q_ads_ (mg g^−1^)	Recovery (%)	Sample	Ref.
MAA	EGDMA	Toluene	7.67–8.65	-	_	[27]
MAA	TRIM	Methylbenzene	29.4	94%	Aqueous samples	[50]
*n*-VCL	NMBA	DMSO	350	-	Blood serum Whole blood Milk	[52]
4-VBA	EGDMA	DMSO	40.1	52.7	River water	[17]
AA	EDGMA	ACN	282.0	-	ACN-H_2_O	[41]
CHI	Au	H_2_O	34.13	98	Aqueous samples	[118]
Allo/*β*-CD	Alginate	H_2_O	635	-	Aqueous samples	[119]
CMD/*m*-PDA	Fe_3_O_4_/CuO	MeOH/H_2_O	1111.1	-	Water	[28]

MAA: methacrylic acid, N-VCL: N-vinyl caprolactam, 4-VBA: 4-vinyl benzoic acid, AA: acrylic acid, EDGMA: ethylene glycol dimethacrylate, TRIM: trimethylpropane trimethacrylate, NMBA: N,N-methylene bis-acrylamide, CHI: chitosan, Allo: allophane, β-CD: β-cyclodextrin, CMD: carboxyl methyl dextrin, m-PDA: m-phenylenediamine.

**Table 8:** Other applications of ciprofloxacin-imprinted polymers.

Monomer	Porogen	Initiator	Solid	Imprinting Technique	Application	Ref.
MAA	MeOH/H_2_O	AIBN	-	Bulk	HPLC	[23,47]
MAA/AM	MeOH/H_2_O	AIBN	-	Monolith	HPLC	[63]
MAA/HEMA	-	AIBN	-	Hydrogel	Drug release	[72]
HEMA/TRIS/AA	CH_3_COOH	UV	-	Hydrogel	Drug release	[71]
HEMA/TRIS/AA	CHCl_3_	UV	-	Hydrogel	Drug release	[70]
LA MAA	MeOH	AIBN	-	Bulk Co-precipitation Emulsion	Drug release	[29]
AM; MAA or 4-VP	MeOH/H_2_O	AIBN	TiO_2_	Co-precipitation	Photocatalyst	[57]
Ti(OH)_4_	EtOH/H_2_O/HCl	-	Fly-ash	Xerogel	Photocatalyst	[46]

MAA: methacrylic acid, AM: acrylamide, HEMA: 2-hydroxyethyl methacrylate, TRIS: (trimethylsiloxy)silane, AA: acrylic acid, 4-VP: 4-vinylpyridine, AIBN: azobisisobutyronitrile.

## Data Availability

The data that support the findings of this study are available from the corresponding author upon reasonable request.
